# Exploring the application boundaries of LLMs in mental health: a systematic scoping review

**DOI:** 10.3389/fpsyg.2025.1715306

**Published:** 2026-02-27

**Authors:** Jinhua Yang, Ting Liu, Yiming Taclis Luo, Tianyue Niu, Patrick Pang, Ao Xiang, Qin Yang

**Affiliations:** 1 The School of Humanities, Tongji University, Shanghai, China; 2 Faculty of Applied Sciences, Macao Polytechnic University, Macao, Macau SAR, China; 3 School of Digital Technology and Innovation Design, Jiangnan University, Wuxi, China; 4 Information Security and Assurance, Northern Arizona University, Flagstaff, AZ, United States; 5 Science in Computer Science, Georgia Institute of Technology, Atlanta, GA, United States

**Keywords:** large language model, LLMS, mental health, mental illness, systematic scoping review

## Abstract

**Background:**

The rapid evolution of large language models (LLMs) has ushered in a new era of artificial intelligence (AI) with unprecedented capabilities in understanding and generating human-like text. This progress has sparked a burgeoning interest in applying LLMs across diverse fields, including healthcare. However, the use of LLMs in mental health remains a complex area that demands rigorous investigation. This systematic scoping review aims to explore the current landscape of LLM applications in mental health, identify key research trends and gaps, and delineate the ethical and practical boundaries, thereby providing a comprehensive framework for future research and clinical practice.

**Methods:**

This study adheres to the PRISMA-ScR (Preferred Reporting Items for Systematic Reviews and Meta-Analyses extension for Scoping Reviews) guidelines. A comprehensive search was conducted across eleven databases (Web of Science, Scopus, PubMed, Medline, CINAHL, Cochrane, ACM Digital Library, IEEE Xplore, ScienceDirect, APA PsycInfo, and Google Scholar). A total of 29 articles were ultimately included in the study.

**Results:**

The application of LLMs in mental health is strategically focused on high-throughput screening and clinical augmentation. The application landscape is characterized by domain specialization, with the focus shifting from general models to specialized BERT models to achieve higher clinical accuracy, particularly for high-prevalence disorders such as depression and high-risk conditions. Data analysis is powered by massive, unstructured corpora from social media, supplemented by the systematic incorporation of structured clinical knowledge. However, significant limitations exist, including insufficient cultural sensitivity in non-Western contexts, challenges in capturing longitudinal patient history, and critical risks related to model value alignment and the generation of clinically misleading information.

**Conclusion:**

LLMs have emerged as sophisticated “Mental Health Agents” with immense potential for providing personalized, knowledge-guided interventions. The core challenge for future development is to transcend basic functionality and achieve clinical rigor. Future research must prioritize deep specialization into psychological models, enhance multimodal integration for comprehensive patient assessment, and urgently develop robust ethical and cultural adaptation frameworks to ensure the models are safe, globally equitable, and reliable for clinical deployment, thereby fulfilling their potential to alleviate the global mental health resource crisis.

## 1 Introduction

Mental health disorders represent a pressing global public health issue, imposing a substantial burden on individuals, families, and societal and economic development (World Health Organization, 2022). According to the World Health Organization (WHO), mental disorders are among the leading contributors to the global disease burden (Vigo et al., 2016). These conditions severely disrupt daily life, occupational functioning, and interpersonal relationships. For instance, major depressive disorder often manifests as persistent low mood, loss of interest, and fatigue, significantly impairing an individual’s ability to study or work (Pan et al., 2019). Generalized anxiety disorder is characterized by uncontrollable worry, tension, and physical discomfort, making it difficult for patients to focus on routine tasks (Rowa et al., 2017). Post-traumatic stress disorder (PTSD), triggered by traumatic events, can lead to intrusive memories, avoidance behaviors, and emotional numbing, markedly reducing social functioning and quality of life (Omopo, 2024). These disorders are not merely collections of clinical symptoms; they exert systematic negative impacts on cognition, emotion, and behavior, ultimately resulting in decreased productivity, increased medical expenditures, social isolation, and functional decline (Fish et al., 2024). Therefore, early identification and effective support for these mental illnesses are crucial to mitigating their long-term individual and societal consequences (Kirkbride et al., 2024).

To address this challenge, the development of innovative, accessible mental health services is foundational for implementing effective interventions and support. Traditional diagnostic models for mental health rely heavily on clinicians’ subjective judgments and structured or semi-structured interviews. While these models are based on clinicians’ extensive clinical experience, they face multiple structural challenges in addressing the global, growing patient population (Stein et al., 2022). These include geographical disparities in professional medical resources, subjectivity and time consumption in the diagnostic process, and barriers to timely and effective care due to stigma, financial constraints, or geographic limitations (Perkins et al., 2018). These challenges collectively drive the urgent need for innovative, scalable, and efficient solutions. In this context, digital technologies, particularly LLMs, are emerging as key drivers of transformation in mental health service systems.

Digital technologies, particularly AI, are widely recognized as transformative forces in reshaping the delivery of mental health services (Liu et al., 2024). Early digital interventions, such as telemedicine platforms and mobile health applications (mHealth), have preliminarily demonstrated their potential to improve service accessibility (Pang et al., 2018). With the rapid advancement of AI technologies, their applications in assisting diagnosis, treatment support, and risk assessment have garnered increasing attention. Early machine learning (ML) models (Luo et al., 2023), such as those based on support vector machines (SVMs) (Sharma et al., 2024) or naive Bayes classifiers (Ibrahim et al., 2024), have shown promise in specific tasks. For instance, natural language processing (NLP) techniques have been used to analyze text sentiment (Li et al., 2025), and speech pattern recognition has aided in diagnostic assessments. However, these models are typically designed for specific tasks, lacking generalizability and the ability to understand complex contexts. This limits their effectiveness in handling the highly unstructured, metaphor-rich, and culturally diverse language data prevalent in mental health.

Recent breakthroughs in AI, particularly the rise of LLMs based on advanced architectures like Transformer, have opened new frontiers for mental health. LLMs (Orrù et al., 2025), trained on massive textual and multimodal data, have acquired robust Natural Language Understanding (NLU), Natural Language Generation (NLG), and contextual reasoning capabilities (Montejo-Raez et al., 2024). Unlike earlier shallow ML models, LLMs exhibit “emergent abilities”—performance gains that exceed what would be expected from mere scaling (Han et al., 2024). This enables them to capture subtle and nuanced semantic associations in human language, making them well-suited to address the complexity and subtlety inherent in mental health (Kumar et al., 2025a). The potential applications of LLMs in mental health diagnostics are multifaceted and are transitioning from proof-of-concept to real-world implementation.

First, LLMs can analyze unstructured text data from social media platforms, online forums, anonymous chat rooms, and personal diaries to identify language biomarkers associated with specific mental disorders (Owen et al., 2024). For example, LLM models can detect subtle linguistic cues indicative of hopelessness, social isolation, or suicidal ideation, providing a non-invasive and efficient method for large-scale mental health risk screening (Gao et al., 2025). Second, LLMs can serve as intelligent assistants for clinicians by processing and integrating complex clinical text data (Lin and Kuo, 2025). For instance, they can analyze electronic health records (EHRs), handwritten clinical notes, and initial consultation transcripts to extract key symptoms, medical history, and behavioral patterns (AlSaad et al., 2024). This information can be synthesized into structured, easily interpretable reports, supporting clinical decision-making and reducing the administrative burden on healthcare providers (Vrdoljak et al., 2025). Third, conversational AI powered by LLMs, such as AI chatbots, can offer patients 24/7 accessible emotional support and behavioral tracking (Chow and Li, 2025). These chatbots engage in natural, empathetic conversations, helping patients express emotions, track mood fluctuations, and implement psychology-based coping strategies (Shi, 2025). The interaction data, anonymized to protect privacy, can be used to monitor patients’ condition changes over time, providing clinicians with long-term, dynamic health data for more timely interventions (Li et al., 2024).

Despite their promise, existing reviews on LLMs in mental health present certain limitations, particularly in systematically exploring their application boundaries and potential risks. Most reviews, such as those by Gautam and Kellmeyer (2025) and Jin et al. (2025), primarily focus on technical performance validation or specific application scenarios, lacking a comprehensive examination of LLMs within broader clinical contexts. These reviews often highlight the performance advantages of LLMs in specific tasks but fail to delve into the ethical dilemmas, technical limitations, and safety risks they may encounter in real-world clinical applications. For example, there is a scarcity of systematic research on critical ethical and technical issues, such as algorithmic bias and data privacy, which are essential for ensuring the safe, fair, and effective deployment of LLMs in mental health. Addressing these gaps is vital to advancing the responsible use of LLMs in this field.

To fill these research gaps, this study aims to conduct a comprehensive, systematic scoping review of the application boundaries of LLMs in mental health. Adhering to the PRISMA-ScR guidelines (Mattos et al., 2023), the study will systematically search multiple key academic databases, rigorously screen relevant literature, and extract and synthesize data. The focus will be on the technical, ethical, and practical boundaries of LLMs in mental health clinical practice, providing scientific evidence and recommendations for technology developers, clinicians, and policymakers. Based on this, the study proposes five research questions:

(1) What are the geographic and temporal trends in the application of LLMs in mental health research?
(2) What are the primary application scenarios and technical types of LLMs in mental health?
(3) Which mental disorders are currently the main focus of LLM research, and what are the data sources and evaluation metrics used?
(4) What are the technical, ethical, and practical limitations and risks of LLMs in mental health?
(5) Based on the analysis of existing research, what are the future research directions and development trends?

## 2 Methods

### 2.1 Search strategy

This study screened relevant papers from eleven databases (Web of Science, Scopus, PubMed, Medline, CINAHL, Cochrane, ACM Digital Library, IEEE Xplore, ScienceDirect, APA PsycInfo, and Google Scholar). The search date was June 27, 2025, and the search terms included “large language model*,” “LLM*,” “generative AI,” “GenAI,” “AIGC,” “AI chatbot*,” “conversational AI,” “natural language processing,” “NLP,” “ChatGPT,” “GPT,” “Bard” OR “Gemini,” “mental health,” “mental illness,” “mental disorder*,” “psychiatric disorder*,” “depress*,” “anxiety,” “schizophrenia,” “bipolar disorder*,” “PTSD,” “suicid*,” and “self-harm,” etc. (see Table 1). The PRISMA-ScR checklist is provided in Appendix AS1.

**Table 1:** Selected databases and search formats.

Database	Search formula
Web of Science	(“large language model*” OR “LLM*” OR “generative AI” OR “GenAI” OR “AIGC” OR “AI chatbot*” OR “conversational AI” OR “natural language processing” OR “NLP” OR “ChatGPT” OR “GPT” OR “Bard” OR “Gemini”) (All fields) AND (“mental health” OR “mental illness” OR “mental disorder*” OR “psychiatric disorder*” OR “depress*” OR “anxiety” OR “schizophrenia” OR “bipolar disorder*” OR “PTSD” OR “suicid*” OR “self-harm”) (All fields) AND (Document Types: Article or Proceeding Paper) AND (Languages: English)
Scopus	(TITLE-ABS-KEY (“large language model*” OR “LLM*” OR “generative AI” OR “GenAI” OR “AIGC” OR “AI chatbot*” OR “conversational AI” OR “natural language processing” OR “NLP” OR “ChatGPT” OR “GPT” OR “Bard” OR “Gemini”)) AND (TITLE-ABS-KEY (“mental health” OR “mental illness” OR “mental disorder*” OR “psychiatric disorder*” OR “depress*” OR “anxiety” OR “schizophrenia” OR “bipolar disorder*” OR “PTSD” OR “suicid*” OR “self-harm”)) AND (LIMIT-TO (DOCTYPE, “ar”) OR LIMIT-TO (DOCTYPE, “cp”)) AND (LIMIT-TO (LANGUAGE, “English”))
PubMed	((((large language model*) OR (LLM*) OR (generative AI) OR (GenAI) OR (AIGC) OR (AI chatbot*) OR (conversational AI) OR (natural language processing) OR (NLP) OR (ChatGPT) OR (GPT) OR (Bard) OR (Gemini)) AND ((mental health) OR (mental illness) OR (mental disorder*) OR (psychiatric disorder*) OR (depress*) OR (anxiety) OR (schizophrenia) OR (bipolar disorder*) OR (PTSD) OR (suicid*) OR (self-harm)))) Filters: Full text
Medline	large language model* OR LLM* OR generative AI OR GenAI OR AIGC OR AI chatbot* OR conversational AI OR natural language processing OR NLP OR ChatGPT OR GPT OR Bard OR Gemini AND mental health OR mental illness OR mental disorder* OR psychiatric disorder* OR depress* OR anxiety OR schizophrenia OR bipolar disorder* OR PTSD OR suicid* OR self-harm
CINAHL	((MH “large language model*”) OR TI (“large language model*” OR “LLM*” OR “generative AI” OR “GenAI” OR “AIGC” OR “AI chatbot*” OR “conversational AI” OR “natural language processing” OR “NLP” OR “ChatGPT” OR “GPT” OR “Bard” OR “Gemini”)) AND ((MH “mental health”) OR TI (“mental health” OR “mental illness” OR “mental disorder*” OR “psychiatric disorder*” OR “depress*” OR “anxiety” OR “schizophrenia” OR “bipolar disorder*” OR “PTSD” OR “suicid*” OR “self-harm”))
Cochrane	large language model* AND mental health
ACM Digital Library	large language model* AND mental health
IEEE Xplore	(“Full Text & Metadata”: large language model* OR LLM* OR generative AI OR GenAI OR AIGC OR AI chatbot* OR conversational AI OR natural language processing OR NLP OR ChatGPT OR GPT OR Bard OR Gemini) AND (“Full Text & Metadata”: mental health OR mental illness OR mental disorder* OR psychiatric disorder* OR depress* OR anxiety OR schizophrenia OR bipolar disorder* OR PTSD OR suicid* OR self-harm) Filters Applied: Conferences Early Access Articles Journals
ScienceDirect	large language model* OR LLM* OR generative AI OR GenAI OR AIGC OR AI chatbot* OR conversational AI OR natural language processing OR NLP OR ChatGPT OR GPT OR Bard OR Gemini AND mental health OR mental illness OR mental disorder* OR psychiatric disorder* OR depress* OR anxiety OR schizophrenia OR bipolar disorder* OR PTSD OR suicid* OR self-harm “Article type: Research articles”
APA PsycInfo	large language model* AND mental health
Google Scholar	large language model* OR LLM* OR generative AI OR GenAI OR AIGC OR AI chatbot* OR conversational AI OR natural language processing OR NLP OR ChatGPT OR GPT OR Bard OR Gemini AND mental health AND mental illness AND mental disorder* AND psychiatric disorder* AND depress* AND anxiety AND schizophrenia AND bipolar disorder* AND PTSD AND suicid* AND self-harm

### 2.2 Data selection and extraction

Records were first imported into reference management software EndNote, where automated screening was performed to remove duplicates and records marked as ineligible based on pre-set criteria, before manual screening. Two independent reviewers (JY and TL) conducted a preliminary screening of the article titles and abstracts based on predetermined inclusion criteria. Any discrepancies between the two reviewers were resolved through consultation with a third reviewer (YL). The inclusion criteria are as follows: (1) Studies specifically targeting LLMs in mental health; (2) Research on LLM technologies for mental health services; (3) Research articles and conference papers; (4) Full-text articles and conference papers published in English. The inclusion and exclusion criteria were designed with a specific focus on the application of LLMs in mental health. This study prioritizes empirical investigations of how LLMs are implemented in mental health services, thereby excluding research that primarily explores individuals’ perceptions, algorithm comparison, attitudes, or opinions regarding LLMs in these domains. Additionally, review articles (e.g., narrative or systematic reviews) were excluded, as they synthesize existing literature rather than present original applications. To ensure comprehensive coverage of diverse research methodologies, the inclusion criteria encompassed qualitative, quantitative, and mixed-methods studies, thereby capturing a holistic range of evidence on the implementation of LLMs in practice. These criteria are summarized in Table 2.

**Table 2:** Inclusion and exclusion criteria.

Inclusion criteria	Exclusion criteria
Research on LLM technologies for mental health services.	Research on technologies other than LLMs in the mental health field.
Research on the application of LLM technology in mental health services.	Research on algorithm comparison, attitudes, views, intentions, benefits, obstacles, impacts, experiences, and usage demands towards LLM technology.
Research-type articles and conference papers.	Review articles, theses, non-academic publications, book chapters, etc.
Full text in English.	Full text in other languages.

### 2.3 Data charting

Based on the review scope methodology guidelines provided by the PRISMA-ScR guidelines (McGowan et al., 2020), a data extraction table was developed. Following a purposive pilot test on five articles selected to represent the diversity of the included studies (e.g., different study designs and LLM applications), the table was refined to ensure comprehensive and relevant data capture. The final data extraction table included the following items: author, year, country, research methodology, type of technology/model, type of issues, mental health application, data source, application performance metrics, limitations, and future research directions. All data were extracted by two independent reviewers. Any disagreements that arose during the data extraction process were resolved through consultation with a third reviewer, ensuring the accuracy and consistency of the extracted information.

### 2.4 Collating, summarizing, and reporting the results

The extracted data were synthesized and analyzed using a narrative synthesis approach to address the research questions of the review. Descriptive findings, such as the distribution of articles by year, country, or research method, were presented through graphs and charts to provide a visual overview of the research landscape. The qualitative findings, particularly those related to the application boundaries, limitations, and future trends, were thematically analyzed and explained through a detailed narrative to provide a comprehensive and nuanced discussion. All explanations and interpretations were verified by all authors to ensure the rigor and validity of the final report.

## 3 Results

**Figure 1:**
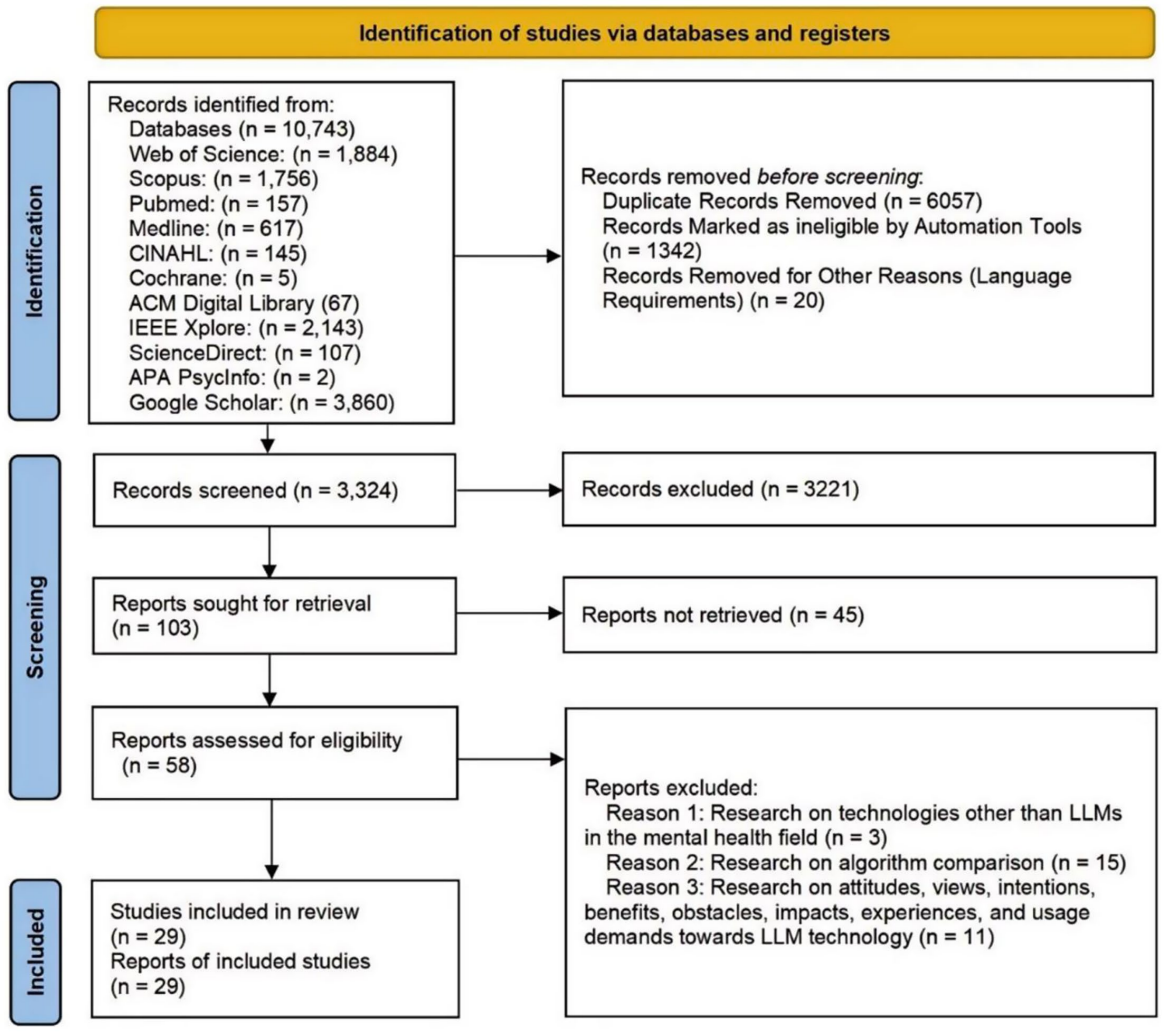
PRISMA flowchart.

A total of 10,743 articles were retrieved through a systematic search, the search process and results as shown in Figure 1. To ensure consistency between the methods, results, and the PRISMA flowchart, we explicitly define the key terms used in the selection process. “Records” refer to all initial items retrieved from the databases. “Reports” or “Articles” refer to unique publications assessed for eligibility. “Studies” refer to the final set of reports that met all inclusion criteria. Two reviewers independently screened the article titles and abstracts, excluding 3,221 articles that were not directly related to the research topic, as well as 20 non-English articles. The term “reports not retrieved,” specifically refers to articles that were identified as potentially relevant during the title and abstract screening but for which the full-text document could not be accessed by the reviewers. Then, the two reviewers conducted a thorough assessment of the remaining 58 articles. These studies were not the focus of this research, so 29 articles were excluded. The reason for exclusion was that the topic did not match the main focus of this study. Ultimately, 29 articles were determined to be included in this systematic review scope (see Table 3).

**Table 3:** Overview of study characteristics.

Author/Year/Country	Methods	Technology/model	Mental issue	Mental health application	Data source	Application performance metrics	Limitations	Future trends
Abdullah et al. 2024 Canada (Abdullah and Negied, 2024)	Quantitative	GPT, BERT	Depression, Anxiety, PTSD, ADHD	Diagnosis, Prediction	167,444 clinical social media posts	F1-score	Cross-validation should be considered to validate the obtained results, validate the hyperparameters, and fine-tune them based on validation results	Future mental disorders from social media data using ML, Ensemble Learning, and LLMs
Al-Otaibi et al. 2025 Saudi Arabia (Al-Otaibi et al., 2025)	Mixed	Transformer, AI Chatbot	Night terror, Depression, Social phobia, panic attacks, Anhedonia, Borderline personality disorder	Support	7,245 tokens from six conversations	Error	AI risks potentially harm vulnerable users; Lack of cross-system comparison	Explore and compare the performance of alternative systems to provide a broader assessment of AI capabilities in this domain
Bartal et al. 2024 Israel (Bartal et al., 2024)	Mixed	Sentence-transformers PLMs, ChatGPT	Childbirth-related post-traumatic stress disorder (CB-PTSD)	Identify	Narratives of length 30 words from 1,295 women	F1 score, sensitivity, specificity, AUC	limitations in analyzing shorter narratives	This textual personal narrative-based assessment strategy employing NLP analysis has the potential to become an accurate, efficient, low-cost, and patient-friendly strategy
Bauer et al. 2024 US (Bauer et al., 2024)	Mixed	LLM embeddings, ChatGPT, eXplainable Artificial Intelligence (XAI)	Suicidality	Understand	2.9 million posts from social media	Mean (SD), Proportion of closest, singular value decomposition	Data time range restrictions and source platform diversity restrictions	Expand the time frame of data collection; explore other web-based platforms; and integrate additional data sources, such as user comments
Belcastro et al. 2025 Italy (Belcastro et al., 2025)	Quantitative	ChatGPT, BERT-XDD model, XAI	Depression	Detect	10,251 tweets + 7,650 posts from social media text	Accuracy, Precision, Recall, F1 score	Labeled data is scarce; Lacks temporal dynamics; Explanatory models may be oversimplified; Cannot replace clinical judgment	Focus on dynamic and longitudinal modeling to capture context and temporal patterns
Cai et al. 2025 China (Cai et al., 2025)	Quantitative	BERT, ChatGPT	General mental health	Information extraction	ChatGPT to generate instances of the task	F1 score	Computational efficiency and scalability constraints; Cross-domain adaptability unverified	Explore a dynamic verification framework; Optimize computational efficiency and scalability, conduct cross-domain validation
Cardamone et al. 2025 US (Cardamone et al., 2025)	Mixed	ChatGPT	General mental health	Prediction	1,000 records from the clinic terms	Recall, F1-score	LLM hallucination and bias risks; Single-label classification limits complexity	Expand coding and classification methods (e.g., multi-label classification), and increase clinical coders
Cremaschi et al. 2025 Italy (Cremaschi et al., 2025)	Mixed	ChatGPT, Retrieval Augmented Generation (RAG) model	General mental health	Decision support	Creation by the ICD-11 classification system	Accuracy, Precision, Recall, F1-score	Lack of patient history integration (e.g., symptom evolution, age, medication history)	Integrate cloud computing; Incorporate temporal evolution, age, and pharmacological information; Focus on comorbidity management and atypical clinical presentations
Dos Santos et al. 2025 Brazil (Dos Santos et al., 2025)	Mixed	BERT, BiLSTM	Depression, Anxiety	Prediction	19.4 million messages from depression data (Twitter/X textual and non-textual data in Portuguese and Reddit textual data in English)	Precision, Recall, F1-score	Discrepancies between model and human judgment (due to clinical caution); Temporal information intentionally omitted	Explore the ethical implications of the model, maintain responsible AI development
Fan et al. 2024 China (Fan et al., 2024)	Quantitative	ChatGPT, AI Chatbot	Depression, Anxiety, Bipolar Disorder, PTSD, Panic Disorder, Eating Disorder	Prediction, Intervention, Suggestion	1,667 entries labeled with psychological disorders from the efaqa dataset, and 1,300 dialogues from the ESConV dataset	Accuracy, Precision, Recall	Ethical and privacy issues regarding the use of data	Future work will explore the model’s ethical implications, maintaining a focus on responsible AI development
Fennig et al. 2024 Israel (Fennig et al., 2025)	Mixed	ChatGPT	Epilepsy, Depression, Anxiety	Assessment	768,504 posts from Reddit’s 21,906 users	Hazard ratios (HRs)	Reddit user base lacks general representativeness; Healthcare environment constraints; Text model’s content understanding variations	Address social stigmatization caused by mental health issues
Hadar-Shoval et al. 2024 Israel (Hadar-Shoval et al., 2024)	Quantitative	ChatGPT	General mental health	Assessment	53,472 individuals across 49 nations	Internal reliability and intercorrelations of Schwartz’s values, Split-half reliability agreement, and confirmatory factor analysis	Small LLM sample size; Difficult to isolate model capabilities from built-in guardrails; Value construct robustness unassessed	Evaluate impact of subtle prompt changes on model values; Validate predictive validity
James et al. 2024 The Netherlands (James et al., 2023)	Mixed	ChatGPT	Severe Mental Illnesses (SMI)	Measurable treatment plan goals	During the five rounds of investigation, these 8 students were given two final goals by LLM	Average scores for both goals in each phase	UI/UX not assessed; No testing with SMI patients or case managers	Recruit SMI patients and case managers to evaluate workflow; Improve UI; Investigate SMI patient attitudes toward privacy and technology
Karamat et al. 2024 Pakistan (Karamat et al., 2024)	Quantitative	Hybrid transformer - MentalBERT, and MelBERT models (CNN)	Depression, Anxiety, Borderline personality disorder (BPD), PTSD	Prediction	40,000 samples from Reddit posts	Accuracy, Precision, Recall, F1-score, ROC, AUC, Loss	Evaluated on a small dataset, failing to capture the full spectrum of disorders/language patterns	Scale up dataset size and diversity for validation
Kharitonova et al. 2024 Spain (Kharitonova et al., 2025)	Mixed	ChatGPT	Depression, ADHD	Content extraction	Formulate 10 questions for each scenario and their corresponding correct answers	Coherence, Veracity, Evidence	Limited content window; Restricted answer search space; Inconsistent multilingual support quality	Experiment with alternative LLMs; Analyze multilingual support; Evaluate text simplifiers; Integrate LLM engine ensembles; Hierarchical information organization; Extend to multimodal systems
Kim et al. 2024 Korea (Kim et al., 2024)	Mixed	ChatGPT	Depression	Support	A four-week field study involving 28 patients with major depressive disorder and five psychiatrists	Coding paired with thematic analysis	Recruitment method impacts generalizability (e.g., young patients, fixed psychiatrists); GPT model updates cause behavioral inconsistency	Compare multiple APP versions and underlying LLMs; Conduct necessary studies with diverse backgrounds
Kumar et al. 2025 UK (Kumar et al., 2025b)	Quantitative	BioGPT, DeBERTa	General mental health	Classification data	DepSeverity consists of 3,553 Reddit posts, SDCNL contains 1895 posts, and Dreaddit comprises 1,191 posts	Accuracy, Precision, Recall, F1-score, AUC, ROC	Reliance on textual data restricts applicability; Reddit data limits cross-cultural/platform generalizability	Evaluate other platforms; Integrate multimodal inputs (e.g., audio, physiological signals); Include real-time human feedback; Explore personalized language modeling
Lozoya et al. 2025 Australia (Lozoya et al., 2025)	Mixed	ChatGPT	Depression, Anxiety	Simulated psychotherapy client interactions	19-question survey	T test	Small sample size of synthetic therapy sessions	Increase professional involvement or session count; Evaluate effectiveness as an educational tool; Explore cross-language and cross-dictionary adaptation
Malhotra et al. 2024 India (Malhotra and Jindal, 2024)	Mixed	BERT, XAI	Depression, Suicidal	Classification, Interpretation	262,922 tweets of data from four datasets	Classification performance evaluation, LIME explanation	Self-reported data lacks manual sanity checks (e.g., for sarcasm, metaphor), leading to false positives	Use data sanity check protocols (manual/automated); Explore XAI applications; Explore multiclass/multilabel/multilingual data analysis and bias detection
Nowacki et al. 2025 Poland (Nowacki et al., 2025)	Quantitative	MentalBERT, MentaLLaMA	General mental health	Classification	3,553 posts from Reddit, 6,850 SMS-like messages, 5,051 records from CAMS, 3029 data from IRF	F1-score	Flan-T5 architecture required additional stabilization layers	Different LLMs are similar to each other. This means that all of these models, despite their different architectures or training methods, achieve a similar level of performance. This indicates their flexibility, comparable quality and ability to process complex data
Park et al. 2024 Korea (Park et al., 2024)	Quantitative	ChatGPT, Zero-shot learning, Medical knowledge graphs	Depression	Information extraction	3,793 PubMed abstracts from BioCreative Datasets, 21,082 documents from Document-Level Relationship Extraction Datasets	F1 score	The limited dataset	Across diverse medical datasets and expanding the types of entities involved
Pavez et al. 2024 Chile (Pavez and Allende, 2024)	Quantitative	BERT, Explainability of Bayesian Networks	General mental health	Diagnosis, Classification	2.3 million data points from social media	Precision, Recall, F1-Score, Support	Method fails to fully capture complex nuances and inherent uncertainties of real-world patient interactions	Deepen collaboration with field professionals for comprehensive assessment; Integrate critical clinical factors (e.g., patient history, hereditary traits)
Radwan et al. 2024 US (Radwan et al., 2024)	Quantitative	nBERT	General mental health	Classification, Recognition	2021 samples	Accuracy, Precision, Recall, F1-score	nBERT generalizability remains a challenge in multilingual and diverse datasets	Explore cross-lingual fine-tuning; Employ transfer learning/cross-domain adaptation; Integrate multimodal data
Shayaninasab et al. 2024 Canada (Shayaninasab et al., 2024)	Quantitative	ChatGPT, AI Chatbot	Depression	Assessment, Support	20 conversational examples (4 examples x 5 depression levels) that were completed with an average of 28 conversation turns, and a total of 562 conversation turns	Depression level	Model cannot classify single input into multiple topics; RAG implementation relies on carefully selected resources	Adopt more sophisticated strategies to address class imbalance
Wagay et al. 2024 India (Wagay and Altaf, 2025)	Quantitative	MentalRoBERTa(6), Capsule Layer, LIME (Local Interpretable Model-agnostic Explanations)	General mental health	Classification	3,553 posts from Reddit	Recall, F1-Score	Platform-specific language remains a challenge; F1/Recall is limited for class imbalance; LIME only offers local, approximate explanations	Adopt more sophisticated strategies to address issues
Wang et al. 2024 China (Wang et al., 2025)	Quantitative	ScaleLLM	Depression	Assessment	Responses from 70,692 participants	Accuracy, Precision, F1-score	Limited capability to process structured data; Rapid evolution of research may outpace LLM updates; Cross-cultural/language adaptability is a major challenge; Lack of real-world clinical validation	Additional language alignment steps; Cross-cultural adaptability; Rigorous testing and validation in practical clinical scenarios
Wu et al. 2024 China (Wu et al., 2024)	Mixed	AI Chatbot	Understanding, Comforting, Evoking, and scaffolding habits	Persuasion	5-week field experiment (N = 25)	Kruskal-Wallis test, Intervention Acceptance Rate	Experimental group limited to young adults; Short field experiment time; Validity/reliability needs improvement; Detection relies on self-reporting; Only considers initial use; GPT-3.5 performance is unstable	Expand sample size and diversity; Improve experimental design; Enhance validity and reliability tests; Explore lighter and more robust LLMs
Zhang et al. 2024 Australia (Zhang T. et al., 2024)	Quantitative	ChatGPT, Zero-shot learning	General mental health	Prediction	Investigation of 150 university students	Zero-shot Mean Absolute Errors	Subjectivity of self-reported datasets; Imbalanced class distributions lead to model bias	Conduct fine-tuning tasks for daily activity-driven models; Increase dataset size or use resampling techniques
Zhang et al. 2024 China (Zhang X. et al., 2024)	Quantitative	AI Chatbot	Depression	Diagnosis, Detection	1,339 conversations from a depression diagnosis dataset	Precision, Recall, F1-score	Inappropriate for real clinical application; Chinese conversational agent; Lacks reliable strategy for optimal training stopping point	Invite wider community participation to enhance the model; Work with different languages

### 3.1 Characteristics of studies

The surge in research on LLMs in mental health is driven by both technological singularity and global public health necessity. All 29 papers analyzed were published or accepted within the extremely narrow timeframe of 2024 to 2025. This trend is a direct result of the revolutionary leaps made by general-purpose LLMs, like GPT-4, since 2023, particularly in complex reasoning, emotional understanding, and generating high-quality, human-like text. This technological capability intersected with the deepening global mental health resource crisis, where hundreds of millions lack effective psychological support. Consequently, the research focus has fundamentally shifted: LLMs are now viewed as key strategic digital assets and are being developed as Digital Mental Health Agents capable of offering clinical decision support, multimodal data integration, and personalized therapeutic interventions, leveraging their characteristics of low marginal cost and high scalability to address the resource gap.

**Figure 2:**
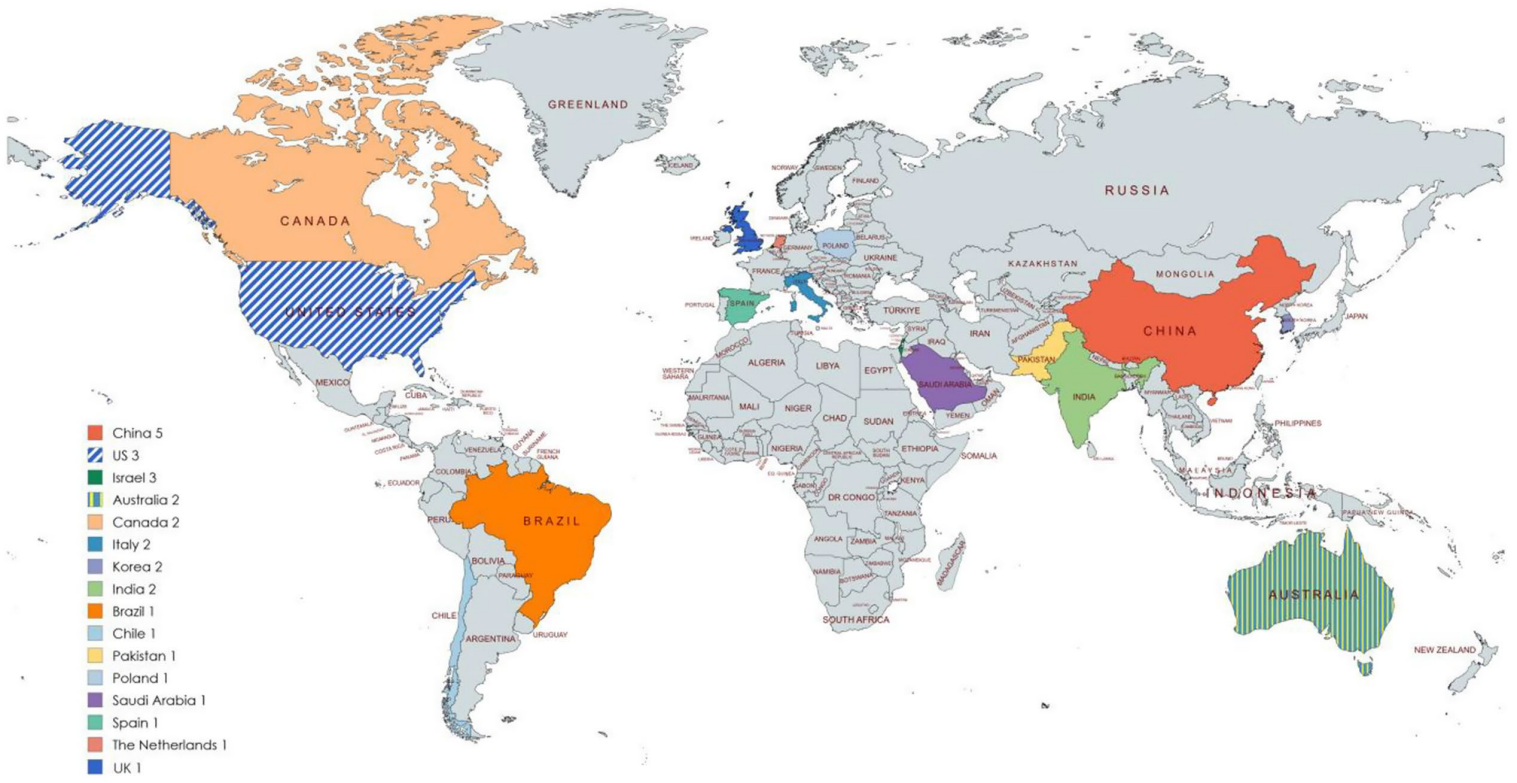
Countries of publication.

The geographic distribution of the research (see Figure 2) (China, the US, and Israel leading the list) clearly illustrates the stratification based on regional economic strength, technological maturity, and public health strategy. China, with 5 papers, leads the world in output, a pattern reflecting its national strategy for basic AI technology localization. Chinese research heavily focuses on developing specialized Chinese foundation models and building knowledge-guided therapeutic applications, aiming to solve the massive mental health resource deficit within its vast population and unique cultural context, emphasizing model professionalism, interpretability, and safety. The US (3 papers) and Israel (3 papers) form the next tier, but with distinct foci: The US leverages its leading data infrastructure and advanced clinical IT systems (EHRs, large-scale social media data) to pursue automated risk prediction and deep integration into clinical workflows. Conversely, Israel, a high-tech innovation hub, focuses on the ethical and psychological depth of AI, concentrating on LLM’s capacity for mentalization, emotional intelligence, and rigorous evaluation of its alignment with human values before widespread deployment.

The middle tier, with 2 papers each from Australia, Canada, Italy, Korea, and India, represents specialized technological penetration. For example, Italy’s work is tailored to its mature but strictly regulated public healthcare system, developing RAG models based on ICD-11 to function as expert clinical decision assistants. Finally, the 8 papers contributed by single nations (including Brazil, Saudi Arabia, the UK, and Spain) often exhibit high pragmatism and cultural adaptation. These regions, frequently facing economic and clinical resource constraints, focus on high-yield, low-cost solutions addressing localized pain points. For instance, Saudi Arabia researches communication errors in Arabic support systems to ensure cross-cultural applicability, while Brazil explores multimodal expert systems integrating text and non-text social data, highlighting a global trend toward diversified, culturally sensitive, and cost-effective LLM deployment against the universal mental health crisis.

### 3.2 Research method of studies

**Figure 3:**
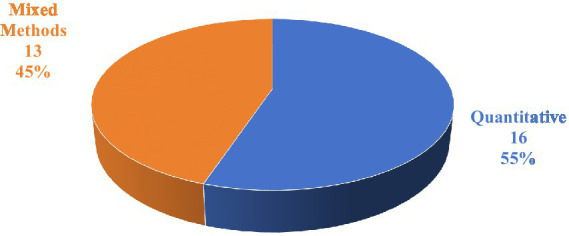
Research method of studies.

The overall methodological distribution in this research reveals a structural balance between technical feasibility and clinical prudence, signifying that the study of LLMs in mental health has advanced to a phase of deep interdisciplinary validation. Out of the total 29 papers analyzed (see Figure 3), 16 papers (approximately 55.2%) utilized a purely quantitative research methodology. This segment is primarily driven by computer science and engineering, focusing on quantifiable technical metrics such as model performance, diagnostic accuracy, data prediction capabilities, and system efficiency, thus establishing the technical feasibility of LLMs as digital healthcare tools. Closely following this, 13 papers (approximately 44.8%) employed mixed research methods, combining quantitative and qualitative approaches. The near-equal split highlights a critical consensus among researchers: in the complex domain of human mental health, technical metrics alone are insufficient. The prevalence of mixed methods demonstrates the essential need to integrate rigorous performance indicators with subjective data on user experience, ethical considerations, cultural adaptability, and clinical acceptance, marking a mature shift in the field from merely asking “what can it do” to “how can it be deployed safely and responsibly.”

### 3.3 LLM technology application landscape in mental health

The comprehensive analysis, drawing from the nine distinct technology combination data points, distinctly charts a sophisticated research landscape in mental health LLM applications characterized by general models as the reference point, domain specialization as the driving force, and clinical trustworthiness as the core architectural principle. ChatGPT, while serving as the most frequently mentioned single model (6 mentions, 16.2%), represents the baseline for general capabilities, and its prominent pairing with Zero-shot Learning (5 mentions, 13.5%) highlights the industry’s successful effort to leverage general LLMs for efficient, low-resource task deployment. Crucially, the dominant research focus has decisively shifted toward vertical domain specialization, evidenced by the overwhelming proportion of dedicated BERT family specialized models, including MentalBERT, MentaLLaMA, BioGPT, DeBERTa, MentalRoBERTa, and nBERT (27 total mentions in combinations), which confirms the community’s critical recognition that achieving the necessary higher clinical accuracy and professional controllability in complex mental health diagnostics requires models trained and fine-tuned on specialized psychological and medical corpora, thereby transcending the limitations of generic language understanding. To ensure safety and confidence in sensitive clinical use cases, Trustworthiness Mechanisms are highly integrated into the architecture: the combination of ChatGPT with the RAG model (3 mentions, 8.1%) is a key strategy employed to ensure factual accuracy by anchoring generated responses to verified knowledge, effectively mitigating the common issue of model “hallucination”; simultaneously, the explicit incorporation of XAI, highlighted by the high-frequency appearance of Explainability of Bayesian Networks (4 mentions, 10.8%) and the complex MentalRoBERTa architecture utilizing LIME (4 mentions, 10.8%), collectively establishes the provision of transparent, traceable decision rationale as an indispensable technical requirement, signaling that the field has unequivocally transitioned into a systematic deployment phase centered on safety, professionalism, and verifiable trust.

### 3.4 Mental health issues

The largest category, “General mental health” (34.5%), establishes the LLM’s role as a large-scale, low-threshold psychological support system, primarily concentrating on non-diagnostic, generalized frameworks for psychological companionship and behavioral intervention, such as providing understanding, comforting, evoking, and scaffolding habits. This reflects the model’s immense value as a universal solution to the scarcity of mental health resources. However, the research focus quickly shifts to specific disorders with high clinical demand and social impact: Depression has emerged as the absolute research core due to its high prevalence, whether as a single illness (20.7%) or as the primary co-morbidity foundation for common conditions like Anxiety, accounting for nearly half of all disorder combinations mentioned. This intense focus on depression signifies the immense potential of LLMs in the early screening, severity assessment, and high-risk prediction of mood disorders. Furthermore, it is noteworthy that research is actively expanding into more complex, trauma-related disorders (e.g., PTSD) and difficult-to-manage co-morbidities like Borderline Personality Disorder (BPD), alongside specialized studies on extremely high-risk issues related to life safety, such as Suicidality (including standalone and combined counts). This necessitates LLMs possessing heightened professional ethics and granular reasoning capabilities. Overall, the application of LLMs in mental health is transitioning from basic text analysis to a profound, specialized tool designed for the precise identification of high-risk individuals, assisting complex clinical diagnosis, and providing professional risk warnings.

### 3.5 Data sources for the LLM mental health

The data sources across these 29 articles reveal a dual driving force in LLM mental health research: the need to quantify vast unstructured data and simultaneously deepen clinical expertise, reflecting researchers’ focus on both high-throughput screening and professional-grade validation. Social media platforms are the unequivocally dominant source, providing a massive corpus of raw, real-world, unstructured linguistic data, with millions of posts and messages from Reddit and Twitter/X, including one dataset contributing up to 19.4 million messages. This heavy reliance on online text is the primary characteristic, aiming to leverage LLMs for high-throughput, real-time detection and risk prediction of mood disorders, particularly depression. Second, the sources underscore a systematic pursuit of professional knowledge and structured data, encompassing knowledge bases created from the ICD-11 classification system, biomedical documents like PubMed abstracts, and clinical records from clinic terms, which are critical cornerstones for building clinical decision support systems and knowledge-augmented LLMs. Furthermore, model validation and optimization are achieved through diverse customized data collection, including large-scale multi-national participant surveys, specific scenario short narratives, and both human-generated task instances and small-scale clinical field studies, signaling a shift from pure text mining towards comprehensive validation of domain-specific customization, ethical alignment, and practical clinical efficacy.

### 3.6 Application performance metrics

The core assessment relies heavily on standard quantitative metrics, with combinations of Accuracy, Precision, Recall, and the F1-score dominating the field, reflecting a primary goal of effective high-throughput screening and detection of mental disorders. Metrics like AUC and ROC further confirm the emphasis on the LLMs’ discriminatory power in risk prediction and binary classification tasks. Crucially, the analysis extends beyond purely technical classification into professional domains: the inclusion of psychometric measures such as Internal Reliability, Split-half Reliability, and Confirmatory Factor Analysis is utilized to ensure the reliability and validity of the psychological constructs being modeled. Furthermore, the appearance of clinical and outcome metrics like Hazard Ratios and T-tests, coupled with the emerging focus on XAI methods (e.g., LIME) and qualitative criteria (e.g., Coherence, Veracity, and Evidence), signals a strategic shift. This transition highlights a commitment to moving LLM applications from simple black-box classifiers to trustworthy, ethically aligned, and clinically interpretable decision-support tools.

## 4 Discussion

### 4.1 Main findings and results of studies

This systematic analysis of research on LLM applications in mental health reveals a field undergoing rapid acceleration within an extremely recent timeframe, driven by unprecedented technological leaps and the urgent global necessity of addressing the immense mental health crisis. This confluence has established LLMs as essential digital mental health agents. Geographically, the research output is concentrated in a handful of technologically advanced and economically strong nations. This includes a major Asian country focusing on specialized national foundation models, localization, and ethical safety, alongside the United States, which leverages its robust data infrastructure and clinical IT systems for automated risk prediction. Another key innovation hub is concentrating on the ethical and psychological depth of AI, specifically evaluating LLMs’ capacity for mentalization and alignment with human values. Methodologically, the field is characterized by an interdisciplinary phase, balancing extensive quantitative research with near-equal attention to mixed methods that integrate technical performance with qualitative data on user experience, ethics, and cultural adaptability. Technologically, while general models serve as a capability baseline, the overwhelming focus has shifted to vertical domain specialization through dedicated, fine-tuned models, a move deemed critical for achieving the necessary clinical accuracy. This specialized architecture is heavily reinforced by trustworthiness mechanisms, such as RAG, to anchor responses to verified knowledge, and the strong integration of XAI methods, ensuring transparent and traceable clinical decision rationales. In terms of application, the largest focus is on providing broad, low-threshold General mental health support, but research is intensely concentrated on common conditions like depression and is actively expanding to complex, high-stakes disorders, including PTSD, BPD, and Suicidality. Finally, the data fueling these advances follows a dual track: it relies heavily on massive, unstructured social media data for real-time high-throughput screening, while also systematically incorporating structured professional knowledge bases and clinical records to build expert-grade decision support systems.

### 4.2 Technical, ethical, and practical limitations and risks of LLMs in mental health

#### 4.2.1 Technical and clinical limitations

The core technical risk of LLMs lies in the deficiency of their clinical accuracy and robustness, particularly when handling high-risk scenarios (Gomes, 2024). For instance, while LLMs have been applied to analyze online discussions to identify high-risk behaviors like suicidal ideation and emotional distress, their precision and reliability in predictive and interventional tasks have not yet met strict clinical standards (Lashgari et al., 2025). A major flaw is the tendency for models to generate “hallucinations,” producing seemingly plausible but false or incorrect clinical information (Kim et al., 2025), which poses a fatal threat to diagnostic decision-support systems like LLMind Chat (Cremaschi et al., 2025). Furthermore, the predictive capacity of LLMs is heavily dependent on the quality of their training data (Yang et al., 2024). Many existing models lack sufficient critical prior knowledge and evidence-based medicine (EBM) data, preventing them from offering deep reasoning and assessments supported by clinical evidence for complex psychological issues (Youngstrom et al., 2017).

#### 4.2.2 Multimodal data challenges

In the realm of multimodal data analysis, such as integrating EEG and physiological signals with text, LLMs show potential, yet they face major technical hurdles (AlSaad et al., 2024). These include data heterogeneity, a lack of interoperability between different sensor systems, and the challenge of establishing a clear clinical correlation between raw sensor data and a person’s mental health status. Effective analysis and reasoning on this disparate data are currently limited (Mezghani et al., 2015).

#### 4.2.3 Practical and cultural barriers

On a practical level, cultural sensitivity represents a significant obstacle. Studies show that even advanced models (like GPT-4o) struggle markedly to identify culturally embedded high-risk narratives (Kazemi et al., 2024). For example, a model’s failure to detect risk signals for filicide-suicide, and its limitations in processing subtle psychological cues specific to certain cultures (Chen et al., 2025). In non-Western linguistic contexts, such as Arabic mental health support inquiries, models like ChatGPT have exhibited clear communication errors, indicating not just a linguistic barrier but a profound lack of understanding of non-mainstream emotional expressions, customs, and help-seeking behaviors (Aleem et al., 2024). For resource-scarce regions (e.g., Africa), LLMs trained on Western principles like Cognitive Behavioral Therapy (CBT) lack cultural resonance with local values, such as limiting user trust, engagement, and effectiveness (Igwe and Durrhiem, 2025).

#### 4.2.4 Ethical and value-alignment risks

The primary ethical risk centers on the model’s value alignment and transparency. The opaque alignment processes of LLMs can unintentionally embed and amplify societal biases, leading to advice that is prejudiced or clinically problematic, potentially harming vulnerable help-seekers (Liu et al., 2023). Concurrently, the over-humanization of conversational AI may blur the lines of the professional therapeutic relationship, leading users to develop unrealistic clinical expectations and dependency (Ngo, 2025). Finally, the handling of highly personal and sensitive mental health data raises acute privacy and security concerns, requiring robust regulatory frameworks to prevent catastrophic data breaches (Kwesi et al., 2025).

### 4.3 Future research directions and development trends

Based on an analysis of existing research, the future direction of LLMs in mental health will be structured around four core pillars: deep specialization, multimodal fusion, ethical framework development, and global cultural adaptability.

#### 4.3.1 Deep technical specialization

The future trend involves moving beyond general-purpose models to develop specialized LLMs dedicated solely to psychological health. These models will be trained on high-quality, evidence-based psychological datasets that include not just single-turn QA but also multi-turn dialogues and real-world case backgrounds augmented by evidence judgment to ensure deep psychological comprehension and evaluation. Research will continue to enhance the performance of unified information extraction, especially for specific languages like Chinese, by introducing components like type verification for more accurate identification of emotions, psychological states, and underlying issues from unstructured text.

#### 4.3.2 Multimodal integration and empathic LLMs

Multimodal integration is poised to be a breakthrough direction, focusing on how to effectively fuse text, physiological signals (e.g., wearable data, EEG), and behavioral health data. Future Physiology-Driven empathic LLMs (Dongre, 2024) will utilize sophisticated techniques like Science-Guided ML (Sharma and Liu, 2022) to automatically extract features from raw physiological data, enabling the model to achieve precise prediction and contextual awareness of the user’s emotional state, thereby providing highly personalized and empathic interventions.

#### 4.3.3 Clinical reasoning and decision support enhancement

Research will prioritize enhancing LLMs’ clinical reasoning capabilities. This includes developing advanced Chain-of-Thought prompting methods to guide models in complex synthesis and reasoning of multi-sensor data, transforming data classification into deep clinical insights for conditions like depression and anxiety. Furthermore, the RAG architecture will be optimized to verify knowledge in real-time from authoritative diagnostic manuals, serving as a core component of powerful clinical decision support systems and ensuring the professional accuracy of diagnostic suggestions and intervention plans.

#### 4.3.4 Ethical frameworks and global cultural adaptation

Future research will place a strong emphasis on model value alignment and cultural fairness. This involves using frameworks like Schwartz’s theory of basic values to conduct continuous, systematic evaluation and correction of LLMs’ intrinsic values, ensuring their decisions align with core human values and avoiding the embedding of harmful biases. Furthermore, research into Africa-centric LLM frameworks will aim to integrate CBT principles with indigenous values like Ubuntu through fine-tuning (Forane et al., 2024), boosting the cultural relevance of LLMs, and providing support that is globally diverse, equitable, and inclusive. Ultimately, the trend is for LLMs to evolve from simple Q&A tools into highly specialized, culturally intelligent “Health Agents” that safely and reliably alleviate the global shortage of mental health resources.

### 4.4 Limitations

The conclusions of this systematic scoping review, which synthesizes the current literature on the boundaries, risks, and future trends of LLMs in mental health, are inevitably subject to the following key limitations. The current evidence base is largely restricted to preliminary exploratory studies and proof-of-concept analyses, significantly lacking the clinical rigor of large-scale randomized controlled trials, thus constraining the assessment of LLM efficacy, safety, and long-term impact with high-level evidence-based medicine validation. Compounding this is the rapid, near-instantaneous evolution of LLM technology, which means the literature analyzed may be quickly outdated, posing a severe timeliness challenge in capturing the newest breakthroughs and emergent risks. Furthermore, a heavy reliance on training data from predominantly English and Chinese contexts results in models with documented deficiencies in cultural sensitivity and language generalizability when dealing with non-Western or minority language narratives, highlighting fundamental issues of cultural equity. Finally, the proprietary and opaque ‘black-box’ nature of many high-performance LLMs restricts systematic scrutiny of their embedded ethical biases and value alignment, severely limiting the reproducibility of academic findings.

## 5 Conclusion

This systematic scoping review aims to systematically explore the boundaries of LLMs in mental health applications, summarizing their core technological pathways, inherent limitations, and future development trends in diagnosis, intervention, and risk prediction. The findings reveal that LLMs have rapidly evolved from simple text analyzers into “Health Agents” capable of integrating multimodal data. Through techniques and the development of specialized models, LLMs are offering novel solutions to alleviate the global shortage of mental health resources. The core strengths of LLMs lie in their advanced language understanding, potential for multimodal fusion, and significant capability to provide personalized, knowledge-guided interventions. However, this review also clearly delineates major challenges facing the field. Technical limitations include the model’s susceptibility to “hallucination,” a lack of clinical evidence-based support, and insufficient robustness and accuracy in high-risk scenarios. Ethical risks are concentrated on the non-transparent value alignment of models, the potential to embed and amplify cultural biases, and the dependency and blurring of the therapeutic relationship caused by over-humanization. Furthermore, the models’ lack of cultural sensitivity in non-Western cultural contexts severely restricts their global scalability and effectiveness. Looking forward, research should focus on: deep specialization, developing psychological professional LLMs based on authoritative psychological and EBM data; advancing cultural equity, developing culturally adaptive LLM frameworks; and establishing regulatory and ethical frameworks to ensure the transparency, trustworthiness, and safe handling of high-risk behaviors by the models. Only through interdisciplinary collaboration and rigorous clinical validation can LLMs be safely and equitably integrated into the mental health service ecosystem to fulfill their immense potential in addressing global psychological distress.
